# Design of a Thiosemicarbazide-Functionalized Calix[4]arene Ligand and Related Transition Metal Complexes: Synthesis, Characterization, and Biological Studies

**DOI:** 10.3389/fchem.2019.00663

**Published:** 2019-10-09

**Authors:** Ehsan Bahojb Noruzi, Mahsa Kheirkhahi, Behrouz Shaabani, Silvano Geremia, Neal Hickey, Fioretta Asaro, Patrizia Nitti, Hossein Samadi Kafil

**Affiliations:** ^1^Department of Inorganic Chemistry, Faculty of Chemistry, University of Tabriz, Tabriz, Iran; ^2^Department of Chemical and Pharmaceutical Sciences, University of Trieste, Trieste, Italy; ^3^Drug Applied Research Center, Tabriz University of Medical Sciences, Tabriz, Iran

**Keywords:** thiosemicarbazide, calix[4]arene, ligand, transition metal complex, antimicrobial, anticancer

## Abstract

In this study, we synthesized a new thiosemicarbazide-functionalized calix[4]arene **L** and its Co^2+^, Ni^2+^, Cu^2+^, and Zn^2+^ transition metal complexes. For characterization several techniques were employed: Fourier-transform infrared (FT-IR), ^1^H nuclear magnetic resonance (NMR), ^13^C-NMR, ^15^N-NMR, correlation spectroscopy (COZY), nuclear Overhauser enhancement spectroscopy (NOESY), electrospray ionization (ESI)–mass spectroscopy, scanning electron microscopy (SEM), energy-dispersive X-ray spectroscopy (EDS), and elemental analysis. To explore the capability of the thiosemicarbazide function hosted on a calix[4]arene scaffold for growth inhibition of bacteria, fungi, and cancerous tumor cells, a series of biological evaluations were performed. For **L**, the antimicrobial tests revealed a higher antibacterial activity against gram-positive *Bacillus subtilis* and a lower activity against gram-negative bacteria (*Escherichia coli* and *Pseudomonas aeruginosa*), whereas the gram-positive *Staphylococcus aureus* shows resistance. All examined metal derivatives show an enhancement of the antibacterial activity against gram-negative *E. coli* bacteria, with a more significant improvement for the Ni^2+^ and Zn^2+^ complexes. MTT assays showed a considerable *in vitro* anticancer activity of Co^2+^, Ni^2+^, and Cu^2+^ complexes against Saos-2 bone cancer cell lines. The activity is ascribable to the inorganic ions rather than calixarene ligand. Hemolysis assay results demonstrated that all compounds have high blood compatibility.

## Introduction

As the third generation of supramolecular hosts, calix[*n*]arenes have attracted considerable attention in a wide range of either demonstrated or potential applications on the basis of their molecular recognition capabilities (Gutsche, 2008; Neri et al., 2016). These applications most notably include direct chemical sensing systems for both ions and neutral molecules (Sliwa and Girek, 2010; Brunetti et al., 2016; Sun et al., 2016; Yeon et al., 2016; Teixeira et al., 2017; Augusto et al., 2018; Cindro et al., 2018; Sarkar et al., 2018), but the host–guest chemistry of calix[*n*]arenes has also been investigated for use in chemical extraction (Du et al., 2018), catalysis (Homden and Redshaw, 2008; Shirakawa and Shimizu, 2018), and various biological/biomedical uses (Perret and Coleman, 2011; Nimse and Kim, 2013; Durso et al., 2016).

The extensive interest in calix[*n*]arenes is correlated with their ease of synthesis and the possibility of successive functionalization of both the upper and lower rims. This offers the possibility to introduce a wide variety of functional groups with different binding properties to fine-tune their supramolecular chemistry. Furthermore, the possibility to functionalize several arms in theory permits them to be multifunctional molecules. Specifically in the case of calix[4]arenes, the high degree of the cavity pre-organization of the cone conformation, combined with this possibility to introduce different functional groups, make them ideal candidates as molecular scaffolds in the design of novel receptors even for metal ions (Sgarlata et al., 2017; Borges et al., 2018; Gaber et al., 2018; Yousef et al., 2018).

Functionalized calixarenes are of particular interest as molecular recognition systems for biomedical applications, and they have been investigated as direct therapeutic agents (William Anthony Coleman, 2010), in drug delivery applications (Rahimi et al., 2018a, 2019), in protein recognition (Doolan et al., 2018), and in imaging applications (Mayer et al., 2018). From this point of view, a potentially interesting strategy that can be applied to calixarenes is functionalization with thiosemicarbazide groups. Thiosemicarbazide groups are functional groups whose derivatives display pharmacological properties that may be further enhanced by incorporation of metal ions (Salah et al., 2018). Thus, both free thiosemicarbazides and their metal complexes have been investigated as anticancer (Güniz Küçükgüzel and Coşkun, 2016; Xie and Peng, 2018; Xie et al., 2018), antiviral (Cihan-Üstündag et al., 2016), and antibacterial (Brahma et al., 2018; Molnar et al., 2018) agents.

The application of calix[4]arenes as receptor of hard metal ions, such as alkaline and alkaline-earth ions, is well-known (Sliwa and Girek, 2010). The incorporation of sulfur atoms introduces the possible coordination of softer transition metal ions, such as Co^2+^, Ni^2+^, Cu^2+^, and Zn^2+^, which are of particular biological interest. The metal complexation can occur through S and terminal N atoms in various ways (Campbell, 1975). For example, the metal complexes of the thiosemicarbazide molecule exhibit square planar (Yang et al., 2006), octahedral (Burrows et al., 1996), square pyramidal (Chiesi Villa et al., 1972), or tetrahedral coordination (Tong et al., 2000) for Co^2+^, Ni^2+^, Cu^2+^, and Zn^2+^, respectively.

The rigid structure of *tert*-butyl-calix[4]arenes means that up to four ligands can be introduced onto the framework through the hydroxy groups in a controlled manner. In general, two thiosemicarbazide groups would be necessary for each calixarene molecule to satisfy the coordination of a single ion. Therefore, the synthetic strategy adopted was to functionalize the 1, 3 positions of the lower rim of the *tert*-butyl-calix[4]arene in cone conformation.

Herein, we report the synthesis and molecular characterization of this novel calix[4]arene-based thiosemicarbazide ligand and its related complexes with some transition metal ions, expressly Co^2+^, Ni^2+^, Cu^2+^, and Zn^2+^. For these compounds, antimicrobial and anticancer activities and biocompatibility were evaluated.

## Experimental

### Materials

All chemical reagents (Merck) and solvents (Merck and Aldrich) were used as purchased without further purification. Human blood was obtained from the Iranian Blood Transfusion Institute. Mueller–Hinton agar (MHA) and Mueller–Hinton broth (MHB) were purchased from Quelab and Merck, respectively. All microorganism strains—*Staphylococcus aureus* (ATCC® 29213™), *Bacillus subtilis* (ATCC® 6633™), *Escherichia coli* (ATCC® 25922™), *Pseudomonas aeruginosa* (ATCC® 27853™), *Candida albicans* (ATCC® 10231™), and *Candida glabrata* (ATCC® 2001™) were provided from Persian Type Culture Collection (PTCC, Karaj, Iran) or Microbiology Department of Drug Applied Research Centre (DARC, Tabriz University, Iran).

### Instrumentation

Fourier-transform infrared (FT-IR) spectra were recorded on a Bruker Tensor 27 spectrometer in the region 4,000–500 cm^−1^ using KBr pellets. Nuclear magnetic resonance [^1^H-NMR, ^13^C-NMR, ^15^N-NMR, correlation spectroscopy (COZY), and nuclear Overhauser enhancement spectroscopy (NOESY)] spectra were taken on a Bruker Spectrospin Avance 400 MHz, Varian 400 and 500 MHz, and Ultra Shield spectrometer with CDCl_3_ solvent. For **L**, Zn^2+^ titration, aliquots of 4 μL of 1 M Zn(NO_3_)_2_·6H_2_O in perdeuterated methanol were added to 0.016 mmol of **L** in 0.6 mL of CDCl_3_ and followed by ^1^H-NMR. NMR spectra were recorded at 25°C after 10 min of equilibration time. Mass spectra were recorded on an ion trap Bruker Esquire 4000 and on a Bruker microTOF-Q, both equipped with an electrospray ionization (ESI) system. Microanalyses were carried out using a Heraeus CHN-O-Rapid analyzer. Melting points were measured on an Electrothermal 9100 apparatus. The morphology characteristic, size distribution, and percentage elemental analysis of samples were conducted via scanning electron microscopy (SEM) (field emission SEM–energy-dispersive X-ray (FESEM-EDX); TESCAN 5001). Prior to examination, samples were mounted onto a metal stub using double-sided carbon adhesive tape and covered with a thin layer of gold, with the aid of a direct current sputter technique (Emitech k450 ×, England). Furthermore, to evaluate the complexation process, elemental analysis by the energy-dispersive X-ray spectroscopy (EDS) technique was performed.

### Synthesis

*p*-*tert*-Butylcalix[4]arene **1** was synthesized using the method published by Gutsche et al. (1981). Compound **2** (5,11,17,23-tetra-*p*-*tert*-butyl-25,27-bis[cianomethoxy]-26,28-dihyroxycalix[4]arene), compound **3** (5,11,17,23-tetra-*p*-*tert*-butyl-25,27-bis[aminoethoxy]-26,28-dihyroxycalix[4]arene), and compound **4** (5,11,17,23-tetra-*p*-*tert*-butyl-25,27-bis[2-isothiocyanoethoxy]-26,28-dihydroxycalix[4]arene) were synthesized using procedures reported by Collins et al. (1991), Zhang and Huang (1997), and Quiroga-Campano et al. (2017), respectively.

#### Preparation of 5,11,17,23-tetra-tert-butyl-26,28-dihydroxy-25,27-bis(thiosemicarbazidoethoxy)calix[4]arene (L)

Compound **4** (0.15 g, 0.18 mmol) was added at room temperature to a stirred solution of hydrazine hydrate (90 μL, 1.8 mmol) in ethanol (5 mL). Stirring was continued for 3 h until a clear yellow solution was obtained. The solvent was evaporated, and the final product was recrystallized from ethanol. Yield 83%. mp: 280°C (dec.). ESI–high-resolution mass spectroscopy (HRMS): [C_50_H_70_N_6_O_4_S_2_+Na]^+^ calcd: 905.4972 *m*/*z*; found: 905.4970 *m*/*z*; FT-IR (KBr, cm^−1^); 583, 798, 875, 937, 1,043, 1,109, 1,200, 1,297, 1,363, 1,476, 1,542, 1,618, 2,871, 2,957, 3,049, 3,437; ^1^H-NMR: (400 MHz, CDCl_3_, tetramethylsilane (TMS), 25°C, δ ppm), 8.35 (s, 2 H; CNHCS), 7.63 (s, 2 H; NNHCS), 7.34 (s, 2 H; OH), 7.06 (s, 4 H; ArH), 6.86 (s, 4 H; ArH), 4.21 (d, 4 H; ArCH_2_Ar), 4.27–4.17 (m, 8 H; OCH_2_CH_2_NCS), 4.12–3.95 (NH2; bs, 4H), 3.37 (d, 4 H; ArCH_2_Ar), 1.28 (s, 18H; t-Bu), 1.00 (s, 18H; *t*-Bu). ^13^C-NMR (101 MHz, CDCl_3_) δ ppm: 183.26 (C-S), 150.22, 149.14, 147.81, 142.45, 132.64, 127.82, 125.99, 125.53, 74.60 (CH_2_O), 44.71 (CH_2_N), 34.20 (C(CH_3_)_3_), 34.02 (C(CH_3_)_3_), 32.05 (ArCH_2_Ar), 31.79 (CH_3_), 31.16 (CH_3_). ^15^N-NMR (50 MHz, CDCl_3_, δ ppm (from CH_3_NO_2_): −274.3 (CH_2_NH); other N's are not detected by ^1^H–^15^N inverse correlation due to proton exchange. Anal. Calcd. for C_50_H_70_N_6_O_4_S_2_ (883.27): C, 67.99; H, 7.99; N, 9.51; found: C, 67.54; H, 8.24; N, 10.04.

#### Synthesis of Metal Complexes

All of the complexes were synthesized as follows.

An appropriate amount of metal salts (0.11 mmol) was dissolved in methanol (5 mL), and the solution was added to a tetrahydrofuran (THF) solution (10 mL) of ligand L (0.11 mmol). The mixture was stirred and refluxed for 24 h, after which the precipitate was filtered and the solvent was eliminated under reduced pressure. The solid obtained was purified by crystallization using THF.

##### Cobalt compound

Co(NO_3_)_2_·6H_2_O salt (0.029 g, 0.11 mmol) was used as the Co^2+^ ion source. A dark brown powder was obtained. Yield: 90%. mp: 230°C (dec.). FT-IR (KBr, cm^−1^), 588, 635, 674, 783, 819, 873, 920, 1,039, 1,120, 1,199, 1,384, 1,482, 1,637, 2,871, 2,958, 3,345. ESI-MS: [C_50_H_69_N_6_O_4_S_2_Co]^+^ calcd: 940.4 *m*/*z*; found: 940.4 *m*/*z*; [C_50_H_68_N_6_O_4_S_2_Co]^+^ calcd: 939.4 *m*/*z*; found: 939.4 *m*/*z*.

##### Nickel compound

Ni(NO_3_)_2_·6H_2_O salt (0.029 g, 0.11 mmol) was used as the Ni^2+^ ion source. A pale green powder was obtained. Yield: 44%. mp: 276°C (dec.). FT-IR (KBr, cm^−1^), 587, 685, 747, 814, 873, 922, 1,038, 1,121, 1,197, 1,238, 1,370, 1,481, 1,564, 1,634, 2,095, 2,958, 3,278. ESI-MS: [C_50_H_69_N_6_O_4_S_2_Ni]^+^ calcd: 939.4 *m*/*z*; found: 939.4 *m*/*z*.

##### Copper compound

Cu(NO_3_)_2_·3H_2_O salt (0.27 g, 0.11 mmol) was used as the Cu^2+^ ion source. A green-brown powder was obtained. Yield: 68%. mp: 270°C (dec.). FT-IR (KBr, cm^−1^), 583, 632, 689, 806, 873, 1,034, 1,108, 1,196, 1,304, 1,367, 1,479, 1,576, 1,635, 2,957, 3,047, 3,397. ESI-MS: [C_50_H_69_N_6_O_4_S_2_Cu]^+^ calcd: 944.4 *m*/*z*; found: 944.4 *m*/*z*.

##### Zinc compound

Zn(NO_3_)_2_·4H_2_O salt (0.026 g, 0.11 mmol) was used as the Zn^2+^ ion source. A pale green powder was obtained. Yield: 83%. mp: 208°C (dec.). FT-IR (KBr, cm^−1^) 581, 674, 783, 1,036, 1,115, 1,198, 1,373, 1,481, 1,635, 2,564, 2,958, 3,270. ^1^H-NMR: (400 MHz, CDCl_3_, TMS, 25°C, δ ppm), 8.49 (s, 2 H; –NHCS), 7.00 (bs, 8 H; ArH), 4.15 (bs, 4 H; ArCH_2_Ar and 8 H; OCH_2_CH_2_NCS), 3.4 (bs, 4 H; ArCH_2_Ar), 1.19 (s, 18H; t-Bu), 1.13 (s, 18H; *t*-Bu). ^13^C-NMR (101 MHz, CDCl_3_) δ ppm: 149.2 (s), 148.7 (s), 148.0 (s), 143.0 (s), 133.2 (s), 127.5 (d), 127.1 (d) 125.6 (d), 34.4 (s, C(CH_3_)_3_), 34.0 (s, C(CH_3_)_3_), 32.6 (t, ArCH_2_Ar), 31.6 (q, CH_3_), 31.2 (q, CH_3_). ESI-MS: [C_50_H_69_N_6_O_4_S_2_Zn]^+^ calcd: 945.4 *m*/*z*; found: 945.5 *m*/*z*.

### Antimicrobial and Antifungal Studies

The minimum inhibitory concentration (MIC) and minimum bactericidal concentration (MBC) of the ligand and related complexes were measured using the microbroth dilution method according to the protocols described by Clinical and Laboratory Standards Institute (CLSI) (Weinstein et al., 2018). Concentration series (15.62–2,000 ppm) of the compounds were prepared in nutrient broth medium. A total of 180 μL of prepared diluted solutions was transferred into sterile 96-well microtiter plates, and then 20 μL of standardized microorganism suspensions was added and mixed gently to get a homogenous suspension. The concentration of the microorganisms was adjusted to 5 × 10^5^ CFU mL^−1^ by 0.5 McFarland solution. Then, the plates were incubated at 37°C for 24 h (Bahlouli et al., 2018). After incubation, turbidity was evaluated to determine bacterial growth, and the dilution with no turbidity (lack of growth) was considered as MIC. Finally, to determine the MBC, samples (5 μL) from tubes in which no growth was observed were cultured in plate (containing MHA medium) and incubated for 24 h at 37°C. In each test, microorganism strain in MHB (without chemicals) and MHB alone (without bacteria) were used as positive and negative growth controls, respectively (Karimi et al., 2018). This method was applied with *S. aureus* (ATCC® 29213™) and *B. subtilis* (ATCC® 6633™) as gram-positive bacteria, *E. coli* (ATCC® 25922™) and *P. aeruginosa* (ATCC® 27853™), as gram-negative bacteria, and *C. albicans* (ATCC® 10231™) and *C. glabrata* (ATCC® 2001™) as fungal strains.

### Cell Culture

MCF-7 cells (human breast cancer cells) and Saos-2 cells (human bone cancer cells) were collected from the Pasteur Institute of Iran, Tehran, Iran, and maintained in RPMI 1640 medium supplemented with 10% fetal bovine serum (FBS) and 1% benzylpenicillin/streptomycin. Further, the MCF-7 and Saos-2 cell lines were maintained at 5% CO_2_ in a CO_2_ incubator at 37°C for 24 h. Cultures were continuously viewed under a microscope to evaluate the quantity of confluence, and the absence of bacterial and fungal contaminants was confirmed. After 90% confluence was reached, the cells were detached by adding trypsin to the flask. The cell suspensions were collected and centrifuged at 1,500 rpm for 5 min and re-suspended in the growth medium for further steps (Rahimi et al., 2017a).

### MTT Assay

To determine the cytotoxic effect of the synthesized ligand and its related metal compounds, a cell viability study was done with the MTT reduction assay. MCF-7 and Saos-2 cells were seeded at a density of 1 × 10^4^ cells/well in 96-well plates. The cells were incubated overnight and treated with **L** or one of the metal complexes at the concentrations of 200, 100, and 50 ppm for 48 h using untreated cells as control. Afterward, the culture media were exchanged with 180 mL of fresh culture media and 20 mL of MTT solution (2 mg mL^−1^) and incubated at 37°C for 4 h. The MTT solution was removed and replaced with 200 μL of dimethyl sulfoxide (DMSO) followed by 20-min incubation time. The absorbance of each well (dissolved formazan crystals) was measured at a wavelength of 570 nm using an enzyme-linked immunosorbent assay (ELISA) reader (Shafiei-Irannejad et al., 2018). The results were given as the mean of three independent experiments. The percentage of viability was calculated by absorbance values using the following formula:

(1) $$\begin{array}{l} \text{Cell viability }\left(\%\right)=\;\frac{A_{\text{sample}}}{A_{\text{control}}}\;\times 100 \end{array}$$

### Hemolysis Assay

#### Blood Collection and Erythrocyte Isolation

Hemolysis assay was performed using fresh human blood. Approximately 5 mL of blood that was stabilized using EDTA was placed into a 15-mL centrifuge tube. Phosphate-buffered saline (PBS) measuring 10 mL was added to wash away blood proteins and serum from the red blood cells (RBCs). The erythrocytes were collected by centrifugation at 4,000 rpm for 10 min at room temperature. The upper layer (plasma) was discarded, and erythrocytes (RBCs) were isolated. The RBCs were washed three times with PBS (pH = 7.4) to obtain a clear supernatant (Rahimi et al., 2018b).

#### Hemolytic Activity and RBC Aggregation

The total isolated RBCs were diluted 10 times with PBS. In each microtube, 0.5 mL of ligand **L** or one of the metal complexes was mixed at different concentrations (62.5, 125, 250, 500, and 1,000 ppm) with 0.5 mL of diluted RBCs and incubated at 37°C for 3 h in an incubator shaker. Diluted RBCs treated with 0.5 mL of water and PBS were used as positive and negative controls with 100% and 0% hemolytic effects, respectively. After incubation, all samples were centrifuged at 4,000 rpm for 5 min. The supernatant was taken out and transferred to a 96-well plate (Rahimi et al., 2018b). An ELISA plate reader was used to measure the released hemoglobin (at a wavelength of 540 nm), and the hemolysis rate was calculated via the following formula:

(2) $$\begin{array}{l} \text{Hemolysis}\left(\%\right)=\;\frac{A_{\text{sample}}-\;A_{\text{negative}}}{A_{\text{positive}}\;-\;A_{\text{negative }}}\;\times 100, \end{array}$$

where *A*_sample_ is the absorbance of the testing sample, and *A*_positive_ and *A*_negative_ are the absorbance of the positive control and the negative control, respectively.

## Results and Discussion

### Development of a New calix[4]arene-thiosemicarbazide Ligand and Related Metal Derivatives

**Figure 1:**
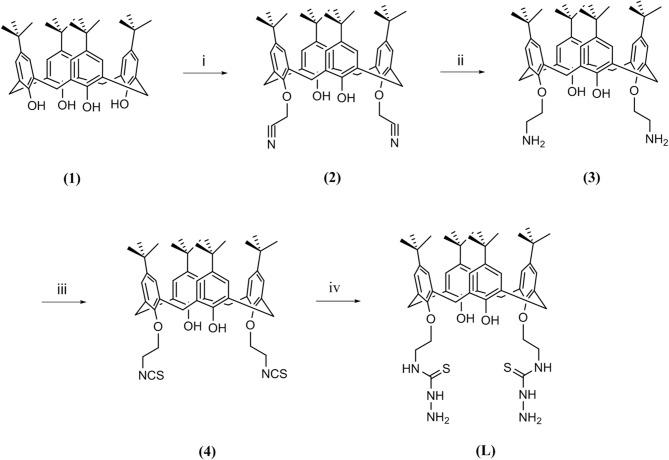
Route followed for the synthesis of ligand **L**; (i) chloroacetonitrile, K_2_CO_3_, NaI, reflux, 7 h; (ii) LiAlH_4_, (0°C), 4 h; (iii) CSCl_2_, BaCO_3_, 24 h; (iv) hydrazine hydrate, room temperature, 3 h.

The synthetic route for the preparation of 5,11,17,23-tetra-*tert*-butyl-26, 28-dihydroxy-25,27-bis(thiosemicarbazidoethoxy)calix[4]arene (**L**) is shown in Figure 1.

Briefly, starting from *p*-*tert*-butylcalix[4]arene (**1**), a couple of hydroxyl groups on the lower rim in 1, 3 alternate positions were transformed to cyanomethoxy by chloroacetonitrile (**2**). The reduction of cyano functional groups with LiAlH_4_ gave the corresponding diamine derivative (**3**). Then, the diisothiocyanate derivative (**4**) was obtained by the reaction of compound **3** with thiophosgene in high yield. Finally, the reaction of compound **4** with an excess amount of hydrazine hydrate in ethanol at room temperature resulted in the new calixarene-thiosemicarbazide derivative (**L**).

Several divalent metal derivatives of **L** (Co^2+^, Ni^2+^, Cu^2+^, and Zn^2+^) were prepared by mixing one equivalent of the metal salt in MeOH with one equivalent of ligand **L** in THF followed by reflux for 24 h.

### Characterization

Several techniques (FT-IR, ^1^H-NMR, ^13^C-NMR, ^15^N-NMR, COZY, NOESY, MS, CHN analysis, SEM, and EDS analysis) were performed to confirm the preparation route of the ligand and metal derivatives.

#### FT-IR

**Figure 2:**
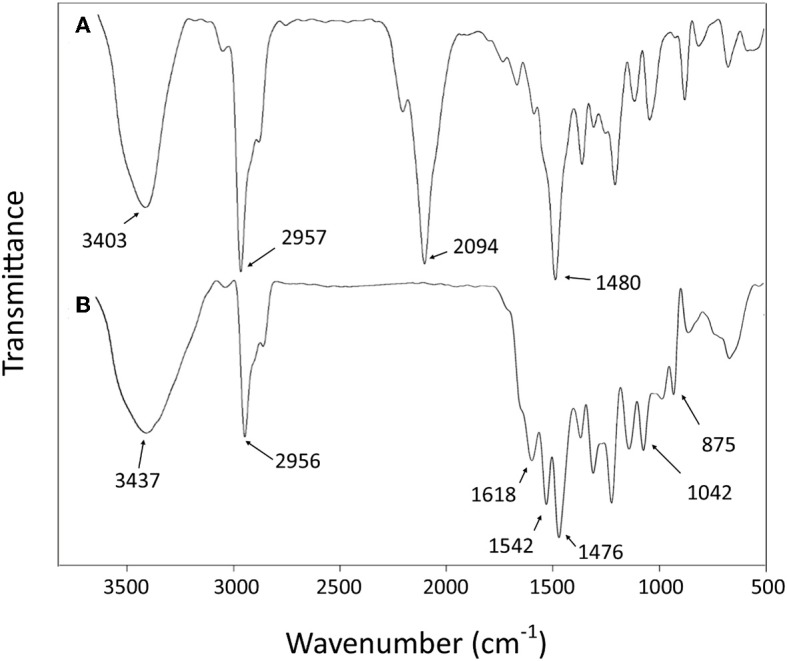
Fourier-transform infrared (FT-IR) spectra of compound **4 (A)** and ligand **L (B)**.

The success of the final step of synthesis is confirmed by the analysis of the FT-IR spectra. The absence of the strong peak at 2,094 cm^−1^ from the spectrum of **L**, assigned to the stretching mode of CN in the NCS group of **4** (Yamamoto et al., 1992), is the most noteworthy feature that indicates the transformation of isothiocyanate functional group (Figure 2). The disappearance of this peak should be accompanied by the appearance of new bands related to stretching and bending vibrations of the formed thiosemicarbazide group, such as the new bands observed at 1,620–1,540 cm^−1^, which can be attributed to the υ(–N–C = S) vibrations (Rao and Venkataraghavan, 1962). The band at 875 cm^−1^ can be attributed to the stretching of the C = S group (Mostafa, 2007). Furthermore, the absence of any bands in the 2,600–2,550 cm^−1^ range assignable to υ(SH) stretching suggests that the CS group remains in thione form (Mostafa, 2007). The expected N–H bond stretching of the thiosemicarbazide group lies within the broad envelope of the peak centered at 3,437 cm^−1^, which also contains the O–H stretching (Silverstein et al., 1991; Rastogi et al., 2002). The other bands, corresponding to C–H, C = C, and C–C vibrational modes of aromatic rings (Baldini et al., 2003; Hernández et al., 2016), are conserved in both **4** and **L** spectra.

The comparison of the FT-IR spectra of **L** and the derivative metal complexes evidences a main feature that supports the metal coordination by **L**. The characteristic double peak of the N–C = S vibration observed for **L** in the 1,620–1,540 cm^−1^ region shifts to higher wave number in all metal derivatives. These differences are summarized in Table 1, and the corresponding spectra are reported in the Supporting Information.

**Table 1:** IR spectral data (cm^−1^) of the ligand and its corresponding complexes in KBr pellets.

**Vibrational mode**	**Frequency (cm^−1^)**				
	**Ligand L**	**Complex Co**	**Complex Ni**	**Complex Cu**	**Complex Zn**
N–C = S	1,618, 1,542	1,637, sh	1,634, 1,564	1,635, 1,576	1,635, sh

*IR, infrared*.

This observation is in agreement with the hypothesis of a metal coordination by **L** for all bivalent ions, with a possible formation of a five-member cycle involving the terminal N-atom and S-atom responsible for the profile change of the N–C = S vibration bands.

#### NMR

**Figure 3:**
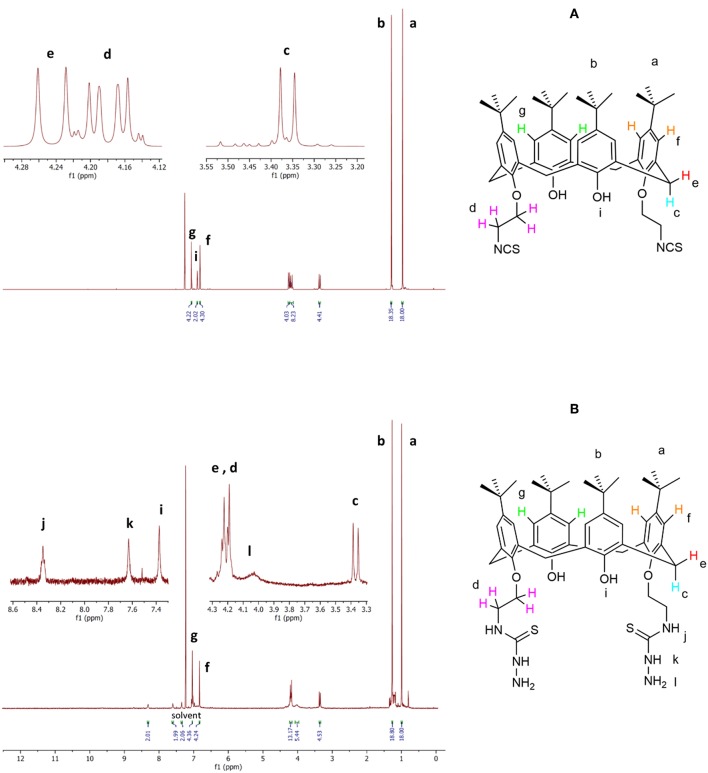
Selected portion of the ^1^H-NMR spectra of compound **4 (A)** and ligand **L (B)**.

The ^1^H-NMR spectra of compound **4** (Figure 3A) show two singlet signals for two sets of 18 *t*-Bu protons (δ 0.97 and 1.29 ppm), two doublet signals (δ 3.36 ppm, 4 protons; and δ 4.25 ppm, 4 protons) assigned to the calixarene –CH_2_- bridging groups, a multiplet peak (δ 4.22–4.14 ppm) corresponding to the eight OCH_2_CH_2_NCS protons, two types of aromatic protons (δ 6.86 ppm, 4 protons; and δ 7.06 ppm, 4 protons) as two singlet signals, and one singlet peak related to two OH protons (δ 6.89 ppm) in agreement with literature data (Quiroga-Campano et al., 2017).

The ^1^H-NMR spectra of ligand **L** (Figure 3B) show analogous signals for the protons that correspond to those in **4**, in addition to a broad singlet for the four terminal NH_2_ protons (δ 4.12–3.95 ppm), a singlet peak attributed to two thiosemicarbazide NH protons (δ 7.63 ppm), and a singlet signal attributed to two NHCS protons (δ 8.35 ppm). The singlet peak corresponding to two OH proton (δ 7.34 ppm) appears at a significantly higher chemical shift than observed for compound **4** (δ 6.89 ppm). The ^1^H-NMR assignment was supported by the H-H and H-C two-dimensional (2D) NMR spectra (see Supporting Information). Thus, these spectra are in agreement with the synthesis of the new calixarene derivative **L**.

**Figure 4:**
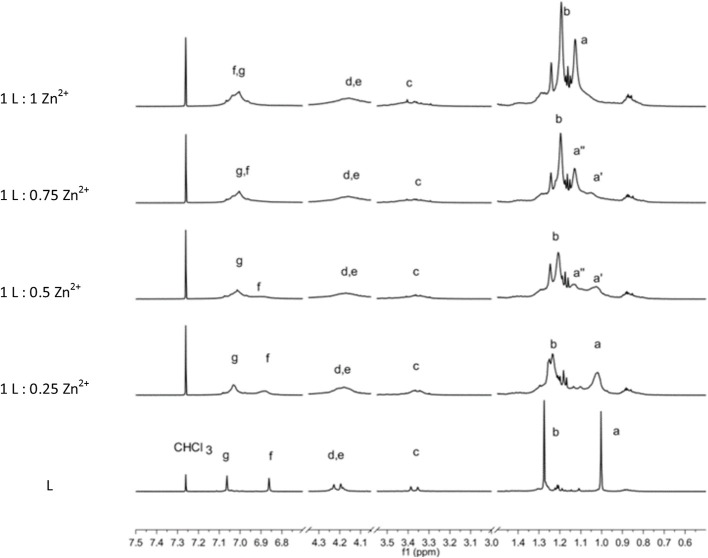
^1^H-NMR spectrum sequence of titration of **L** by Zn^2+^ ions. The bottom spectra of **L** are scaled by a factor of 0.2.

The complexation of the metal ions was investigated by NMR only for the diamagnetic Zn^2+^ ion. The addition of a Zn(NO_3_)_2_ solution to the **L** sample provokes a general broadening and shift of ^1^H-NMR signals depending of the type of protons involved. In particular, the signals of protons of the *p*-*tert*-butyl of phenyl groups functionalized by the thiosemicarbazide arms (a) and the corresponding aromatic hydrogens (f) are the most affected (Figure 4).

Owing to the overall broadening of the signals, the two strongest singlets due to sets of 18 *tert*-butyl protons (a, b) are the best indicators to follow the complexation process. The stepwise addition of Zn^2+^ ions causes a downfield shift of the (a) protons, whereas the (b) protons are shifted upfield. In particular, the (a) signal shows a slight shift upon the first addition of a Zn^2+^. Then with the second addition of Zn^2+^ ion (**L**:Zn^2+^ ratio = 1:0.5), a split of the (a) signal becomes evident. The two signals (a′ and a″) 0.1 ppm apart that have approximately equal intensity can be attributed to species with unbound and bound Zn^2+^ ions, respectively. When the titration reaches the 1-to-1 ratio, the (a′) signal disappears in favor to the (a″) signal of the Zn^2+^ complex. Contemporarily, a significant downfield shift of the aromatic proton (f) is observed. This titration experiment clearly shows the coordination properties of the **L** ligand and the formation of a 1-to-1 stoichiometric complex.

#### ESI-MS

The HRMS of ligand **L** showed molecular ion peaks corresponding to the sodium adduct of the **L** ligand (Supporting Information). The most intense peak of 905.4790 *m*/*z* perfectly agrees with a compound with formula C_50_H_70_N_6_O_4_S_2_ (theoretical [M + Na]^+^ equal to 905.4792 *m*/*z*) and confirms the proposed structure for **L**.

Evidence of formation of the complexes was obtained by ESI-MS (Supporting Information). All spectra show peaks related to multiple species along with evidence for formation of the metal ion complexes. The spectrum for the Co adduct shows the presence of Co^2+^ bound by a deprotonated **L** ligand ([C_50_H_69_N_6_O_4_S_2_Co]^+^, *m*/*z* 940.4) superimposed with an amount of Co^3+^ species bound by a doubly deprotonated **L** ligand ([C_50_H_68_N_6_O_4_S_2_Co]^+^, *m*/*z* 939.4). It should be noted that the deprotonation of two hydroxy groups of calix[4]arene is consistent with formation of an octahedral coordination environment ideal for complexation of Co^3+^ species. The spectrum of Ni derivative, in which an ion is usually involved in octahedral coordination, shows the clearest evidence of metal complexation of those investigated. The most intense peaks are related to the mono-deprotonated **L** ligand coordinated to Ni^2+^ ion ([C_50_H_69_N_6_O_4_S_2_Ni]^+^, *m*/*z* 939.4). In the case of Cu^2+^ and Zn^2+^, the MS show a series of peaks related to the [C_50_H_69_N_6_O_4_S_2_Cu]^+^ (944.4 *m*/*z*) and [C_50_H_69_N_6_O_4_S_2_Zn]^+^ (945.5 *m*/*z*) complexes, respectively. For both species, the isotopic distribution shows some small discrepancy in the intensity with respect to the calculated distributions.

#### SEM

**Figure 5:**
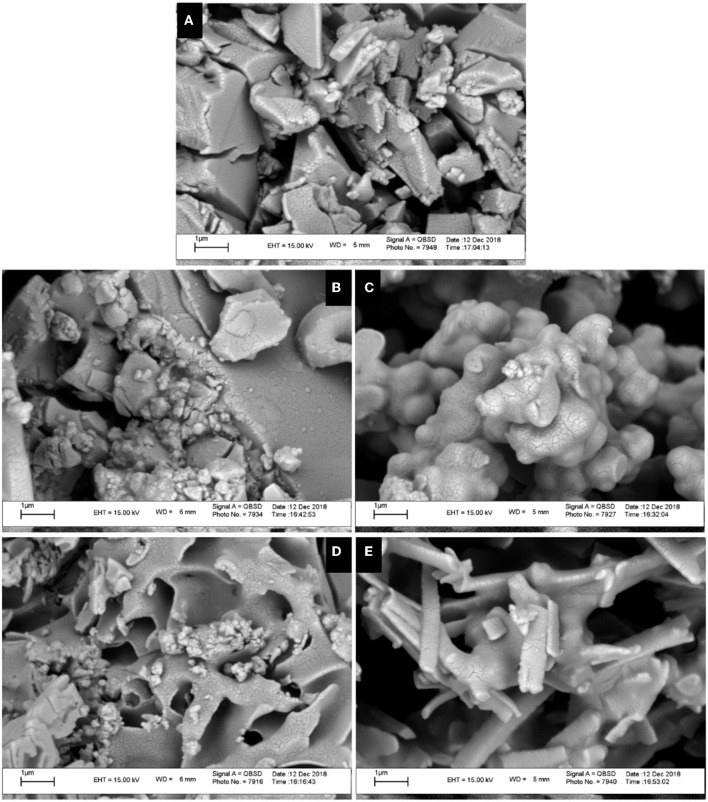
Scanning electron microscopy (SEM) images of ligand **L (A)** and complexes **Co (B)**, **Ni (C)**, **Cu (D)**, and **Zn (E)** at the scale of 1 μm.

The morphological characteristics of the solid-state aggregation of ligand **L** and its metal derivatives were evaluated using a SEM by applying 15-kV electron acceleration voltage. Figures 5A–D shows the SEM images of ligand **L** and of its metal derivatives at 20× magnification. In comparison with **L**, each metal derivative shows a different morphology of the aggregation state, with a more crystalline tendency in the case of the Zn derivative (Figure 5E).

#### EDS

**Figure 6:**
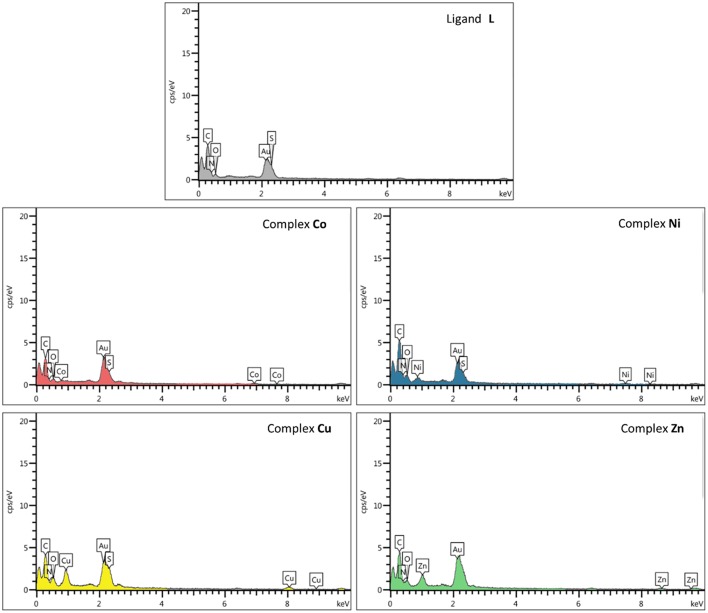
Scanning electron microscopy–energy-dispersive X-ray (SEM-EDX) elemental analysis of ligand **L** and complexes **Co**, **Ni**, **Cu**, and **Zn**.

EDS or EDX was used to semi-quantitatively measure chemical elements of compounds. As indicated by the EDS chemical maps, the presence of Co, Ni, Cu, and Zn metals is confirmed in the corresponding metal derivative samples (Figure 6). The analysis of the data suggests that the percentage of metal ions in the Cu^2+^ and Zn^2+^ samples is somewhat higher than that observed for the Ni^2+^ and Co^2+^ derivatives.

### *In vitro* Antimicrobial Activity and Antifungal Evaluation

The biological properties of thiosemicarbazide derivatives have been studied extensively in recent years by different research groups (Liesen et al., 2010; Plech et al., 2011; Shebl et al., 2013; Rane et al., 2014). Herein, the *in vitro* antimicrobial and antifungal activities of the synthesized compounds (**L** and complexes of **Co**, **Ni**, **Cu**, and **Zn**) were evaluated using the microbroth dilution method against the aforementioned standard strains. None of the investigated compounds show a measurable antifungal activity. However, as shown in Table 2, the free ligand **L** has an effective antibacterial activity against gram-positive *B. subtilis*, whereas the gram-positive *S. aureus* was resistant to it. A lower activity was observed for both investigated gram-negative pathogens (*E. coli* and *P. aeruginosa*). The cobalt complex showed a moderate antibacterial activity against all of the bacteria except *B. subtilis* that showed resistance. The antibacterial properties against the gram-negative strains were significantly enhanced upon coordination of nickel to the thiosemicarbazide ligand, whereas no variations are observed for gram-positive bacteria. Specifically, in the case of *E. coli* strain, the MIC and MBC values show four-fold and two-fold decreases, respectively. The coordination of copper metal to the ligand improved the MIC and MBC values against *S. aureus* and *E. coli* strains. On the other hand, the MIC values against *B. subtilis* and *P. aeruginosa* were not affected by this metal addition, whereas the MBC values against these two strains were enhanced. In comparison with **L**, the zinc complex improved the antibacterial activity toward *S. aureus* and both gram-negative bacteria. In the case of *E. coli* and *P. aeruginosa* strains, the MIC values show eight-fold and two-fold decreases, respectively. On the other hand, the MBC values against all of the microorganisms (except *P. aeruginosa*) were improved significantly, with a 16-fold decrease against *E. coli*. Overall, all compounds have some antibacterial activities against gram-negative strains, and some of them showed an antibacterial activity against gram-positive bacteria. The decreased MIC and MBC values of metal derivatives are consistent with the possibility that they disturb the respiration process of the bacterial cell, blocking the synthesis of proteins, which restricts growth of the microorganism (Dharmaraj et al., 2001).

**Table 2:** Antibacterial activity of synthesized compounds against different microorganisms in with the microbroth dilution method.

**Microorganisms**		**Gram positive**		**Gram negative**	
		***Staphylococcus aureus***	***Bacillus subtilis***	***Escherichia coli***	***Pseudomonas aeruginosa***
**MIC**	Ligand **L**	–	31.25	250	62.50
	Complex **Co**	250	–	125	125
	Complex **Ni**	–	31.25	62.50	31.25
	Complex **Cu**	250	31.25	125	62.50
	Complex **Zn**	500	125	31.25	31.25
	Gentamicin^*^	0.12	2	0.5	2
**MBC**	Ligand **L**	–	–	500	–
	Complex **Co**	–	–	500	500
	Complex **Ni**	–	–	250	–
	Complex **Cu**	250	31.25	250	250
	Complex **Zn**	500	125	31.25	–
	Gentamicin^*^	0.25	2	0.5	2

The antibacterial activity is expressed as the MIC and MBC (ppm). MIC, minimum inhibitory concentration; MBC, minimum bactericidal concentration.

* *Gentamicin used as standard control for bacteria*.

#### MTT Assay

**Figure 7:**
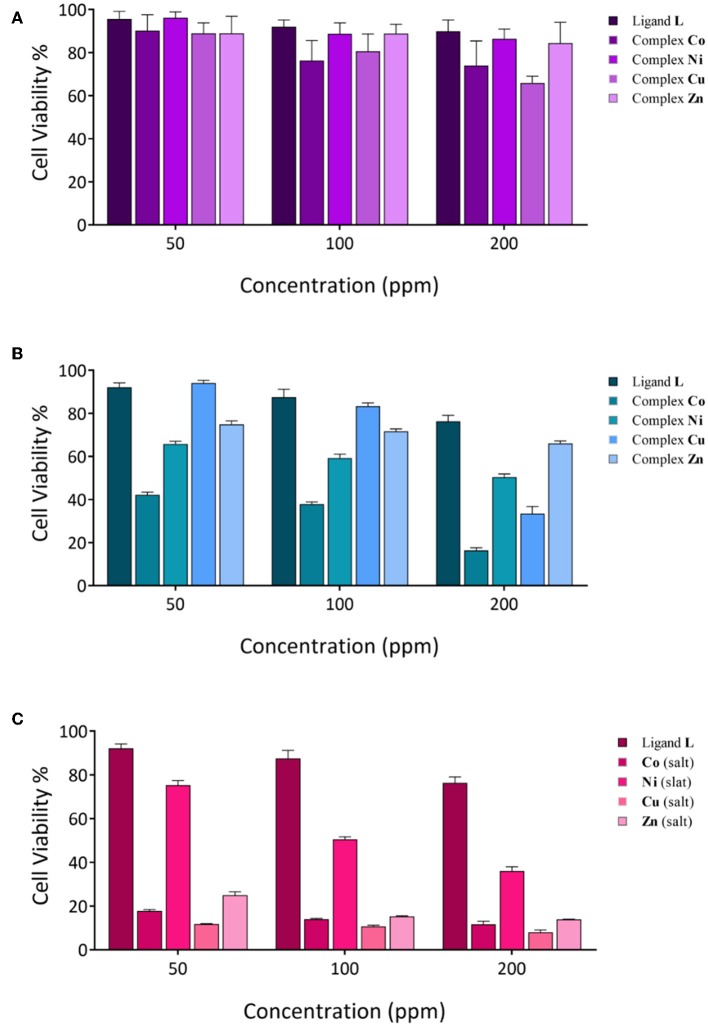
Cytotoxicity evaluation of synthesized compounds at various concentrations against cancer cell lines; **(A)** MCF-7 cell lines; **(B,C)**, Saos-2 cell lines.

According to previous studies, the anticancer activities of calixarene-based compounds are related to their enzyme inhibition potential (Cherenok et al., 2006, 2012), inhibiting tumor angiogenesis (Dings et al., 2006) and DNA replication of cancer cells (Consoli et al., 2007). The cytotoxicity of the ligand and complexes was studied against the MCF-7 and Saos-2 cell lines by MTT assay. All of the compounds were dispersed in water (and 10% DMSO) and diluted with cell culture medium to reach three required concentrations (50, 100, and 200 ppm). The cytotoxicity impact on cell growth is shown in Figure 7. In general, all of the compounds showed a very low antiproliferative activity against MCF-7, whereas for the Saos-2 cells, a more significant dose-dependent antiproliferative activity was observed with exclusion of **L** and the **Zn** derivative. The **Co** derivative showed an excellent anticancer activity against the Saos-2 cell line even at a lower concentration (42.13%), although the most effective toxicity is revealed at a higher concentration (16.34%). At a higher concentration, the activities of **Ni** and **Cu** derivatives are also evident, with inhibition concentrations (IC_50_) over 48 h of 200 and 170 ppm, respectively. However, control assays performed with the corresponding inorganic salts show that the activity is mainly ascribable to the inorganic component rather than the organic calixarene component. Actually, the **L** ligand appears to protect the cells against the inherent cytotoxicity of the bivalent ions present in the inorganic salts of these metals.

#### Hemolysis Assay

**Figure 8:**
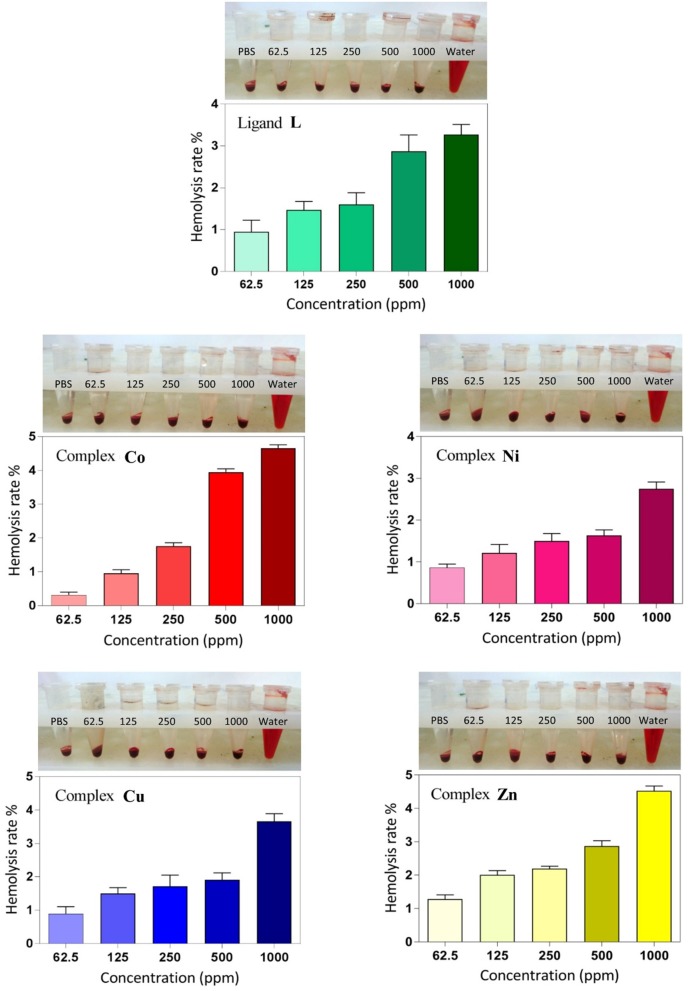
Hemolysis rate of human red blood cells (HRBCs) in the presence of the synthesized samples and the visual observation of hemoglobin in the supernatant at different concentrations (ppm).

Hemolysis assay was performed to examine the biocompatibility and the cytotoxic effect of ligand and complexes on RBCs at different concentrations. As shown in Figure 8, dose-dependent hemolytic effects of the prepared compounds on RBCs were observed, and the results confirm that there are only slight hemolytic effects from the compounds at high dosages. RBCs were also treated with PBS and deionized water as negative and positive controls, respectively. With regard to the results, at the highest concentration investigated (1,000 ppm), the lowest hemolytic activity was observed for the **Ni** derivative (2.6%), whereas at the lowest concentration investigated (62.5 ppm), the lowest hemolytic activity was observed for the **Co** derivative **(**0.3%). Therefore, on the basis of biocompatibility of the various compound investigated, the **Ni** and **Co** complexes could be the best choices for biological applications in higher and lower concentrations, respectively. Also, the hemolytic activity of the ligand itself is very low (3.18%) and <4.5%, which is an acceptable threshold for hemolytic activity (Rahimi et al., 2017b).

## Conclusion

In this study, we designed and synthetized a new calix[4]arene-based thiosemicarbazide molecule, and we used it as a quadri-dentate ligand to prepare a series of transition metal complexes. The characterization with multiple techniques (FT-IR, ^1^H-NMR, ^13^C-NMR, ^15^N-NMR, COZY, NOESY, ESI-MS, SEM, EDS, and elemental analysis) demonstrated the success of the synthesis of the target ligand, **L**. The ability of **L** to complex metal ions was demonstrated by ^1^H-NMR titration experiments with the diamagnetic Zn^2+^ ions. On the basis of the known biological properties of thiosemicarbazide derivatives, we performed antimicrobial and anticancer evaluations against human cancer cell lines and biocompatibility studies on **L** and the series of its metal complexes to investigate Co^2+^, Ni^2+^, Cu^2+^, and Zn^2+^. **L** showed a higher antibacterial activity against gram-positive *B. subtilis* and a lower activity against gram-negative bacteria (*E. coli* and *P. aeruginosa*), whereas the gram-positive *S. aureus* shows resistance. All metal derivatives show an enhancement of the antibacterial activity against gram-negative bacteria (except for the Co^2+^ and Cu^2+^ derivatives for *P. aeruginosa*), with a more significant improvement for the Ni^2+^ and Zn^2+^ complexes. The anticancer activities of all compounds against the MCF-7 cell line were not relevant, even if the activities of the **Co** and **Cu** complexes slightly improved at a higher concentration. On the other hand, the MTT assay appeared to show a significant anticancer activity of Co^2+^, Ni^2+^, and Cu^2+^ complexes against Saos-2 cell line. However, control assays show that this activity is mainly ascribable to the metal ions rather than the organic calixarene component. Hemolysis assays demonstrated no significant hemolysis rate for all of the compounds even at higher concentrations.

## Data Availability Statement

All datasets generated for this study are included in the manuscript/Supplementary Files.

## Ethics Statement

All biological studies were carried out under approved protocols with the ethical committee of Tabriz University of Medical Sciences (National Institutes of Health Publication No. 85-23, revised 1996).

## Author Contributions

EB and MK: substantial contributions to the conception or design of the work, or the acquisition, analysis or interpretation of data for the work. BS: research have performed under his supervision. SG and NH: substantial contributions to the analysis or interpretation of data for the work. FA: performed the NMR titrations of metal complexes. PN: purified some samples and helped for interpretation of NMR spectra. HK: designed and confirmed antibacterial part of research.

### Conflict of Interest

The authors declare that the research was conducted in the absence of any commercial or financial relationships that could be construed as a potential conflict of interest.
