# Supplementary material for: Design of a Thiosemicarbazide-Functionalized Calix[4]arene Ligand and Related Transition Metal Complexes: Synthesis, Characterization, and Biological Studies
Source: Front Chem. 2019 Oct 9;7:663. doi: 10.3389/fchem.2019.00663 (PMC6794423; doi:10.3389/fchem.2019.00663)
Supplement: Supplementary file 1 [file Data_Sheet_1.docx]

**Supporting Information**

Design of a thiosemicarbazide functionalized calix[4]arene ligand and related transition metal complexes: Synthesis, characterization and biological studies

Ehsan Bahojb Noruzi,^a^ Mahsa Kheirkhahi,^a^ Behrouz Shaabani,*^a^ Silvano Geremia,^b^ Neal Hickey,^b^ Fioretta Asaro,^b^ Patrizia Nitti,^b^ Hossein Samadi Kafil^c^

^a^ Faculty of Chemistry, Department of Inorganic Chemistry, University of Tabriz, Tabriz, Iran

^b^ Department of Chemical and Pharmaceutical Sciences, University of Trieste, Trieste, Italy

^c^ Drug Applied Research Center, Tabriz University of Medical Sciences, Tabriz, Iran.

| **Table of Content:** | **Pages** |
| --- | --- |
| **FT-IR Spectra** | S2-S4 |
|  |  |
| **NMR Spectra** | S5 – S12 |
|  |  |
| **ESI-MS** | S13 – S22 |
| **MIC/MBC values** | S23 |
| **References** | S24 |

**FT-IR Spectra of L and L-Co, L-Ni, L-Cu, L-Zn derivatives**

**
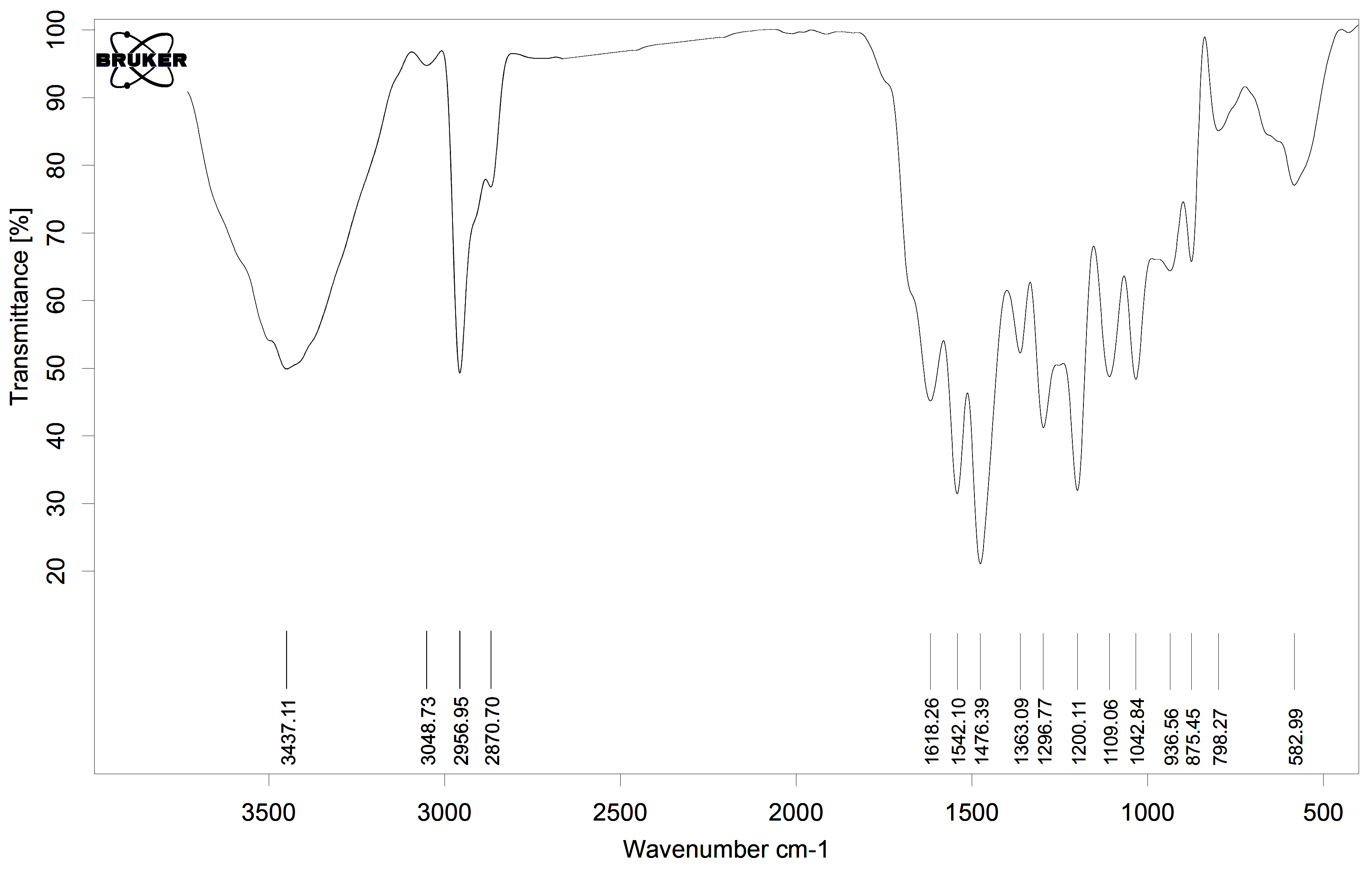
**

**Figure S1:** FT-IR spectrum of **L** (KBr pellet).


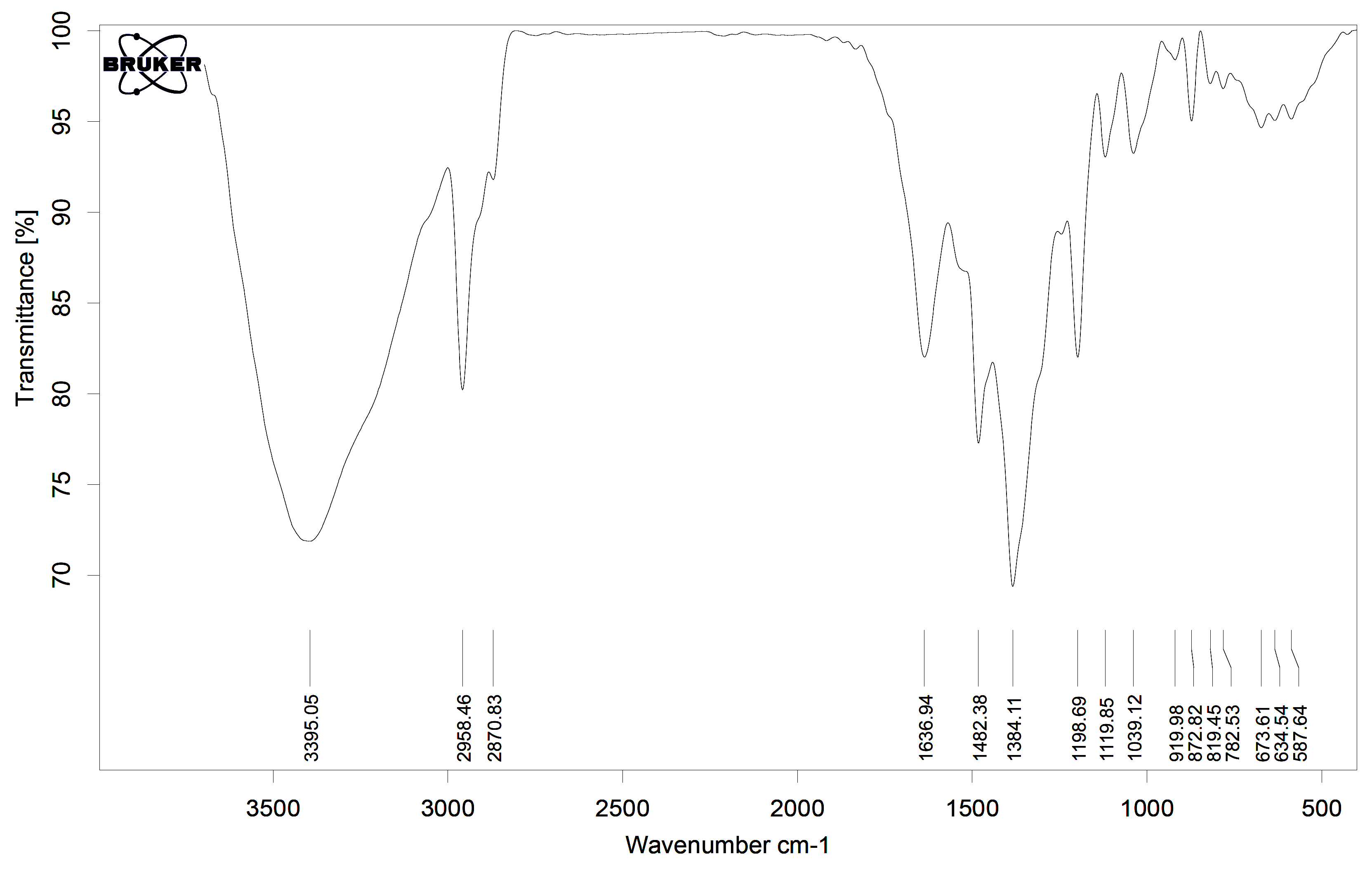


**Figure S2:** FT-IR spectrum of **L-Co** derivative (KBr pellet).


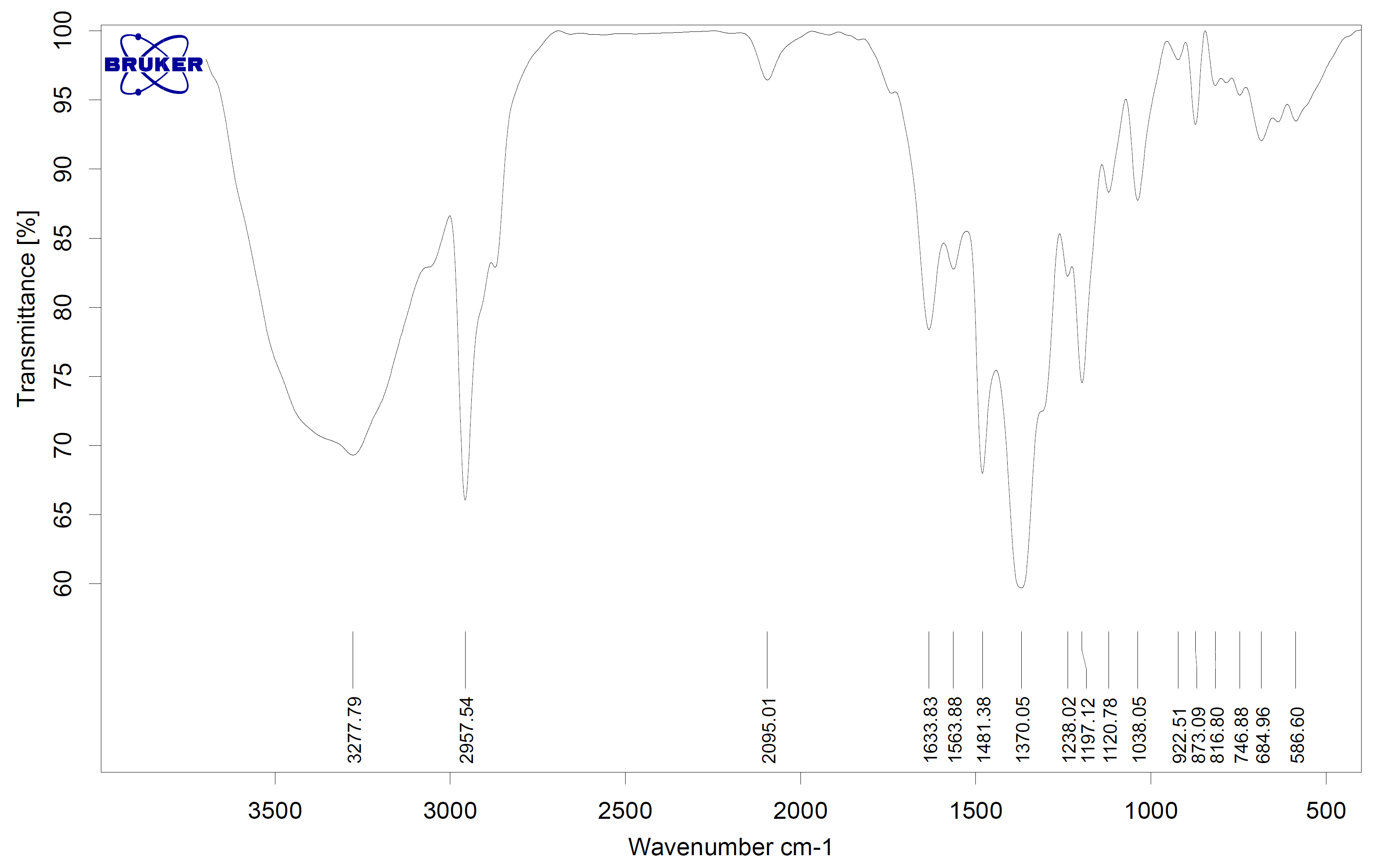


**Figure S3:** FT-IR spectrum of **L-Ni** derivative (KBr pellet).

**
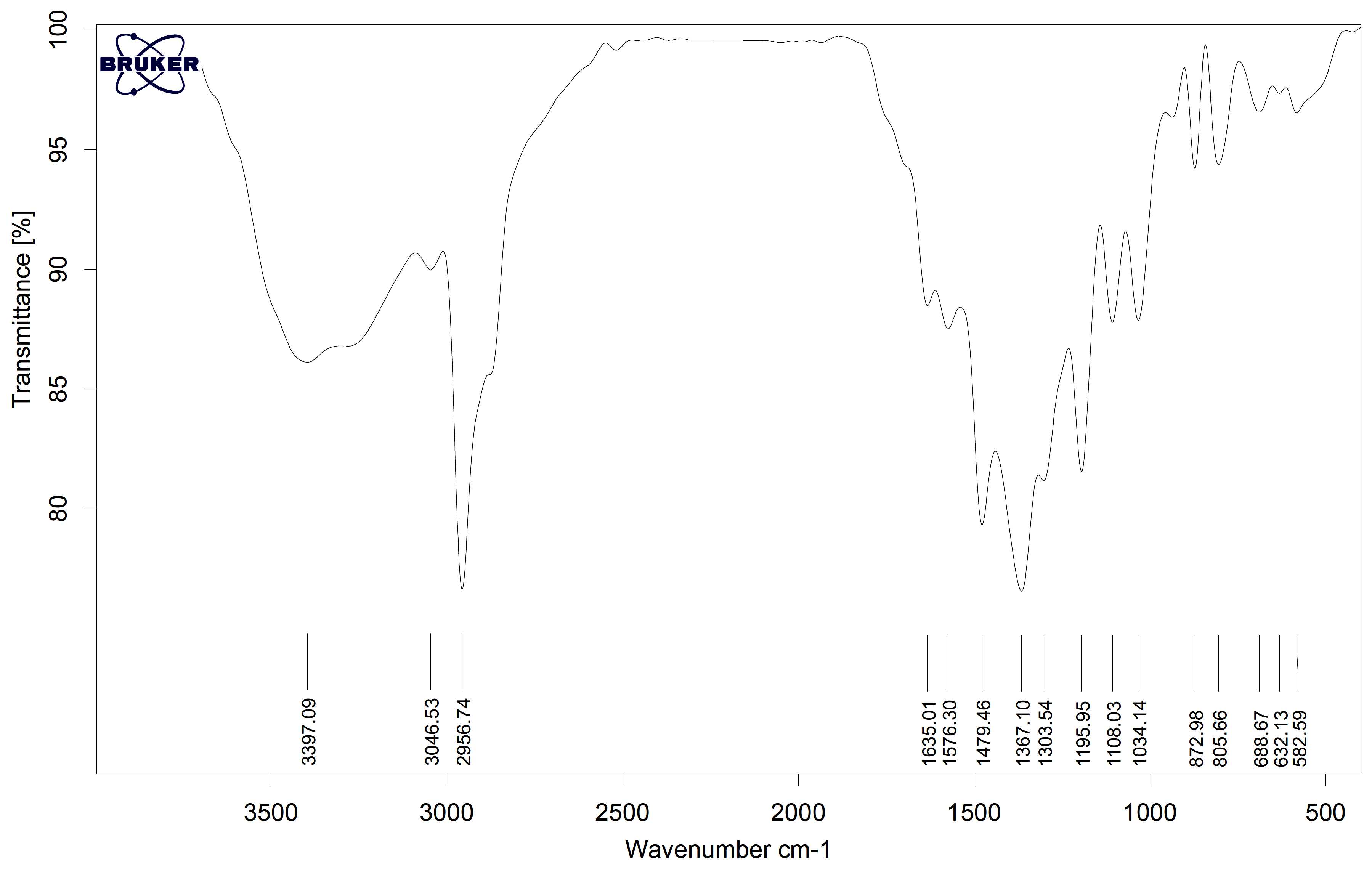
**

**Figure S4:** FT-IR spectrum of **L-Cu** derivative (KBr pellet).


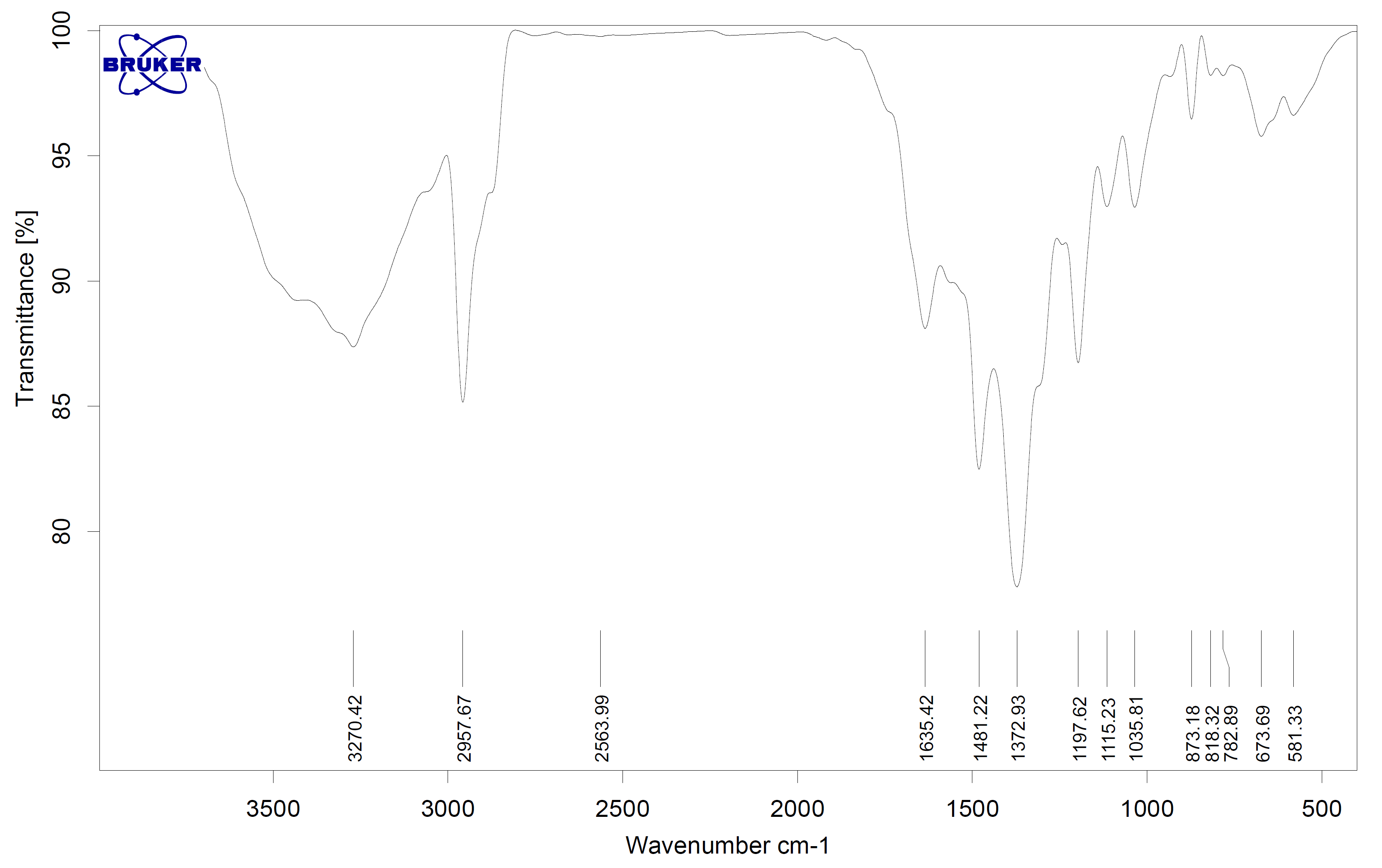


**Figure S5:** FT-IR spectrum of **L-Zn** derivative (KBr pellet).

**NMR Spectra**

**Figure S6:** HH-COSY spectrum of L.

**Figure S7:** HC-HSQC spectrum of L with spectral editing: CH_3_ and CH red, CH_2_ blue.

**Figure S8:** ^1^H-^15^N-HMBC Spectrum of L.


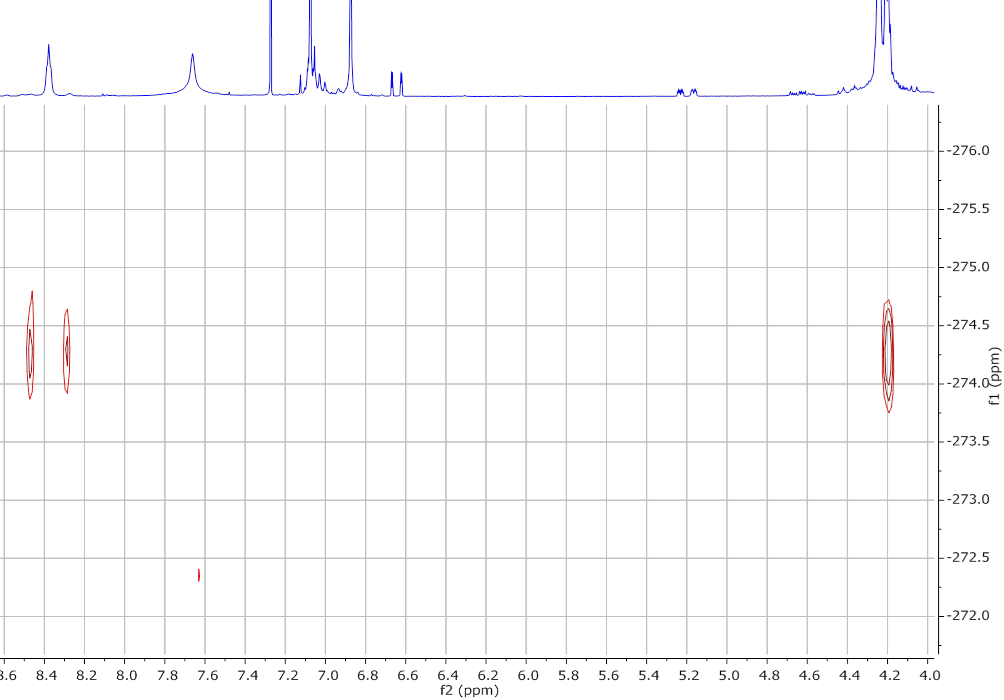


**Figure S9:** Enlargement of the ^1^H-^15^N-HMBC Spectrum of L, which shows the cross peaks between the ^15^N nucleus resonating at -274 ppm (shift referred to CH_3_NO_2_) and the CH_2_-N protons via a ^3^J(H,N coupling) and with the directly bound proton by the ^1^J(^15^N,^1^H), which is responsible for the 90 Hz splitting observed in the f2 dimension. This shift value is in agreement with literature data for thiosemicarbazide thioamide nitrogens.^1^ The signal of the other ^15^N could not be observed by the inverse experiments using CDCl_3_ as solvent because of the fast exchange of the directly bound protons and the lack of close protonated carbons long range correlation.^2^

**Figure S10:** NOESY spectrum of L complexing Zn^2+^ in a solution of L added by with on addition of an equivalent amount of Zn(NO_3_)_2_

**Figure S11:** Enlargement of the above NOESY spectrum: cross peaks between tBu protons and aromatic protons. For the assignment see text.

 **Figure S12:** ^13^C NMR spectra of L before (black) and after addition of an equivalent amount of Zn(NO_3_)_2_ (blue)

**Figure S13:** Region of tert-butyl carbon signals with the quaternary carbons resonances at higher ppm.

**ESI-MS of L and Co, Ni, Cu, Zn derivatives**

**
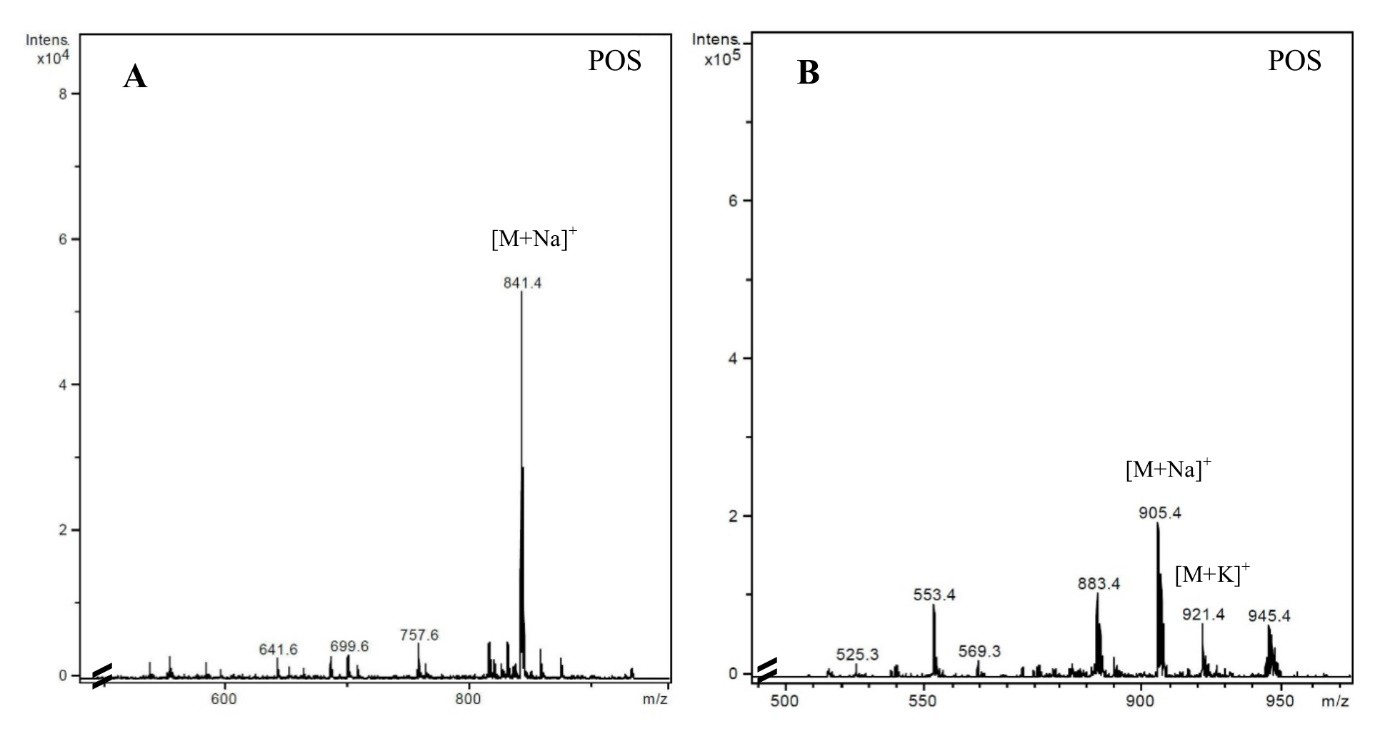
**

**Figure S14 :** Positive-ion ESI-Mass spectrums of compounds **4** (A) ([M+Na]^+^) and ligand **L** (B) ([M+Na]^+^).and ([M+K]^+^).


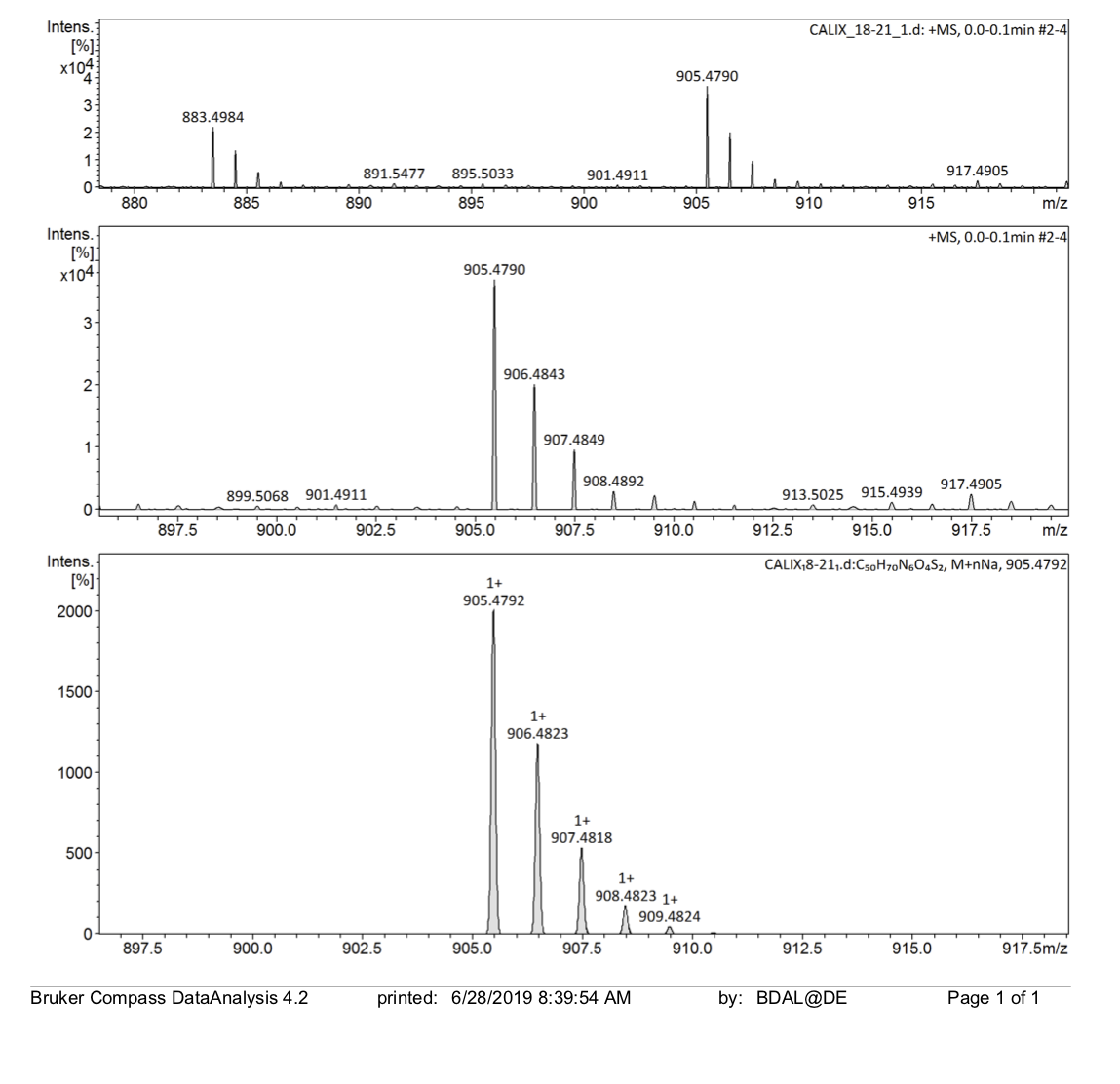


**Figure S15:** High resolution mass spectra (HRMS) of **L**. Exact masses were calculated by enviPat Web 2.2 (http://www.envipat. eawag.ch/index.php).^3^

**
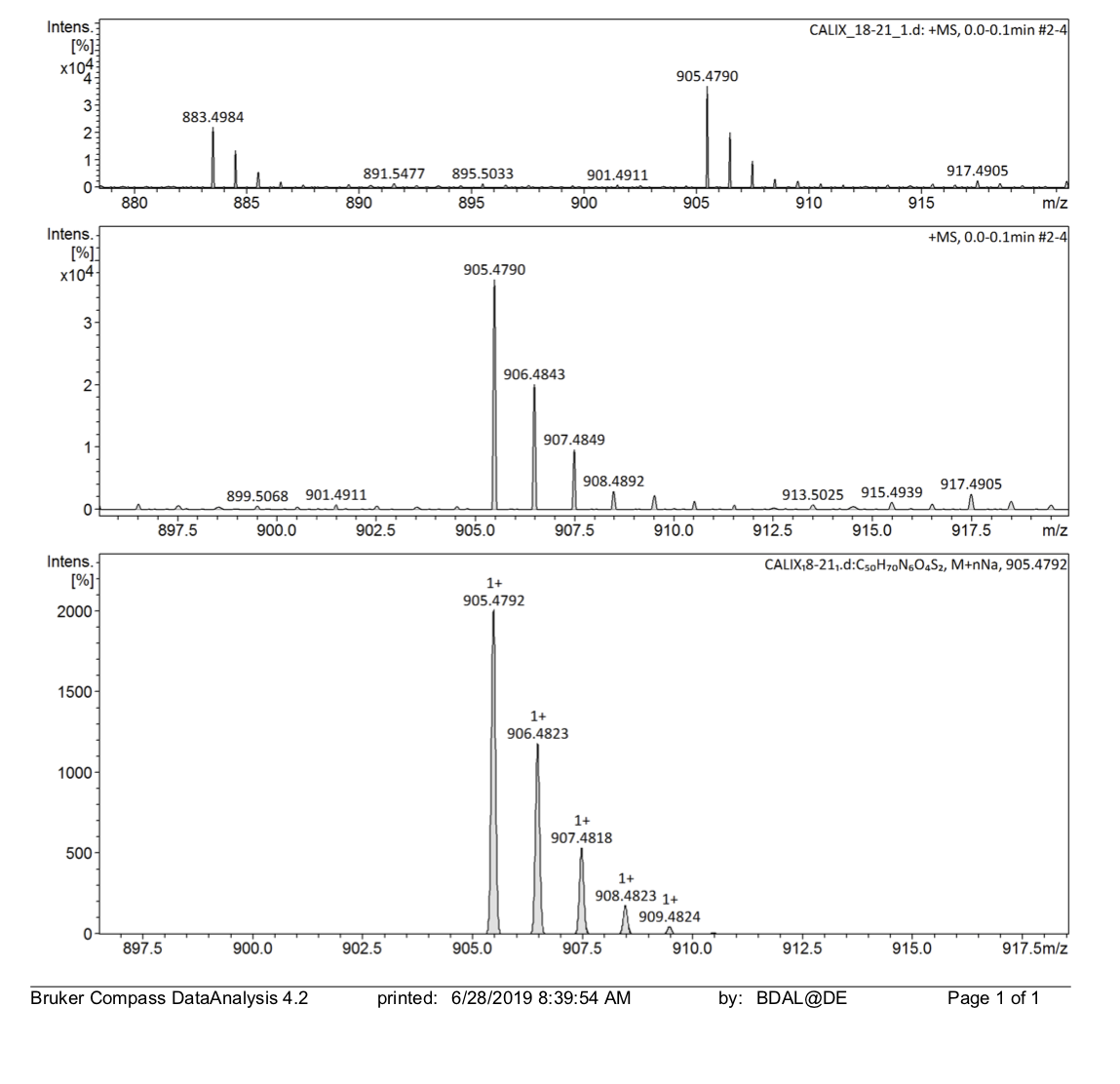
**

**Figure S16:** Positive-ion ESI-Mass spectrums of Co-L derivative.

(b)

**Figure S17:** Calculated (a) and observed (b) isotopic distributions for a mixture containing 0.65 [C_50_H_69_N_6_O_4_S_2_Co]^+^ (m/z 940.4) and 0.35 [C_50_H_68_N_6_O_4_S_2_Co]^+^ (m/z 939.4). Masses were calculated by enviPat Web 2.2 (http://www.envipat. eawag.ch/index.php).^3^

**Figure S18:** Positive-ion ESI-Mass spectrums of Ni-L derivative.


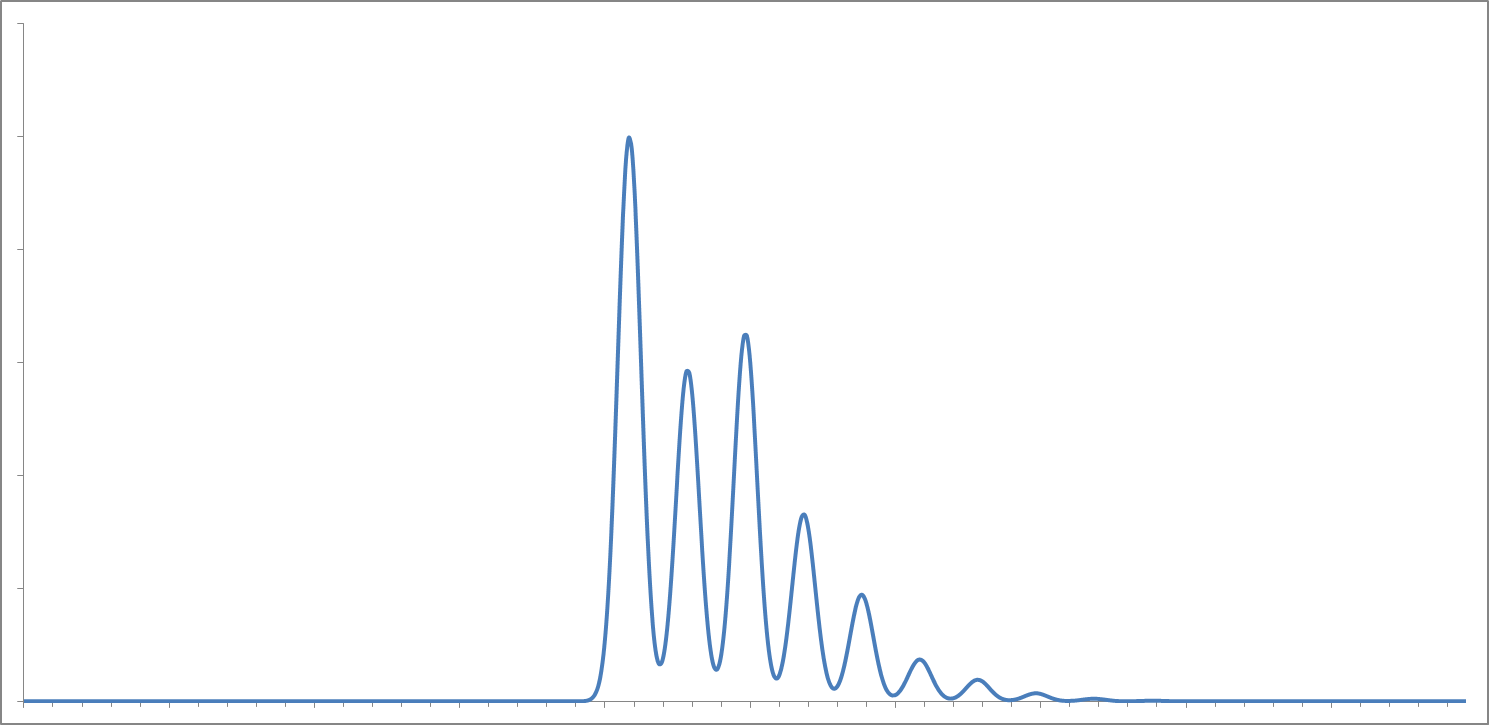


(a)

(b)

**Figure S19:** Calculated (a) and observed (b) isotopic distributions for [C_50_H_69_N_6_O_4_S_2_Ni]^+^ (m/z 939.4). Masses were calculated by enviPat Web 2.2 (http://www.envipat. eawag.ch/index.php).^3^

**Figure S20:** Positive-ion ESI-Mass spectrums of Cu-L derivative.

(b)

**Figure S21:** Calculated (a) and observed (b) isotopic distributions for [C_50_H_69_N_6_O_4_S_2_Cu]^+^ (m/z 944.4). Masses were calculated by enviPat Web 2.2 (http://www.envipat. eawag.ch/index.php).^3^

**Figure S22:** Positive-ion ESI-Mass spectrums of Cu-L derivative.

(b)

**Figure S23:** Calculated (a) and observed (b) isotopic distributions for [C_50_H_69_N_6_O_4_S_2_Zn]^+^ (m/z 945.5). Masses were calculated by enviPat Web 2.2 (http://www.envipat. eawag.ch/index.php).^3^

**Table S1** Antibacterial activity of metal salts against different microorganisms in

micro-broth dilution method. The antibacterial activity is expressed as the MIC and MBC (ppm).

| **Microorganisms** | | **Gram positive** | | **Gram negative** | |
| --- | --- | --- | --- | --- | --- |
|  |  | [**S. aureus**](https://www.google.com/search?safe=active&rlz=1C1GCEA_enIR853IR853&q=s+aureus&spell=1&sa=X&ved=0ahUKEwjTkdiRr_LiAhWtyKYKHUO1DooQkeECCCooAA&cshid=1560838131868423) | [**B. subtilis**](https://en.wikipedia.org/wiki/Bacillus_subtilis) | **E. coli** | **P. aeruginosa** |
| MIC | Co salt | 1000 | 1000 | 2000 | 2000 |
|  | Ni salt | 2000 | 2000 | 2000 | 1000 |
|  | Cu salt | 2000 | - | - | 1000 |
|  | Zn salt | 2000 | 1000 | - | 1000 |
| MBC | Co salt | 2000 | 2000 | 2000 | 2000 |
|  | Ni salt | - | - | - | - |
|  | Cu salt | - | - | - | - |
|  | Zn salt | 2000 | 2000 | - | - |

**References**

1. Arendse, M. J.; Green, I. R.; Koch, K. R., Synthesis and spectral studies of platinum complexes of para-substituted 4-phenylthiosemicarbazides. *Spectrochimica Acta - Part A: Molecular and Biomolecular Spectroscopy* **1997,** *53* (10), 1537-1545.

2. Yavari, I.; Roberts, J. D., Nitrogen‐15 nuclear magnetic resonance spectroscopy. Differential rates of N-H proton‐exchange reactions of hydrazine‐carbothioamide and 4‐methylhydrazine‐carbothioamide in dimethyl sulfoxide. *Organic Magnetic Resonance* **1980,** *14* (1), 61-64.

3. Loos, M.; Gerber, C.; Corona, F.; Hollender, J.; Singer, H., Accelerated isotope fine structure calculation using pruned transition trees. *Analytical Chemistry* **2015,** *87* (11), 5738-5744.
